# A critical role of mevalonate for peptidoglycan synthesis in *Staphylococcus aureus*

**DOI:** 10.1038/srep22894

**Published:** 2016-03-10

**Authors:** Yasuhiko Matsumoto, Jyunichiro Yasukawa, Masaki Ishii, Yohei Hayashi, Shinya Miyazaki, Kazuhisa Sekimizu

**Affiliations:** 1 Laboratory of Microbiology, Graduate School of Pharmaceutical Sciences, The University of Tokyo, 7-3-1 Hongo, Bunkyo-ku, Tokyo 111-0033, Japan

## Abstract

3-hydroxy-3-methyl-glutaryl-CoA (HMG-CoA) reductase, a mevalonate synthetase, is required for the growth of *Staphylococcus aureus*. However, the essential role of the enzyme in cell growth has remained unclear. Here we show that three mutants possessed single-base substitutions in the *mvaA* gene, which encodes HMG-CoA reductase, show a temperature-sensitive phenotype. The phenotype was suppressed by the addition of mevalonate or farnesyl diphosphate, which is a product synthesized from mevalonate. Farnesyl diphosphate is a precursor of undecaprenyl phosphate that is required for peptidoglycan synthesis. The rate of peptidoglycan synthesis was decreased in the *mvaA* mutants under the non-permissive conditions and the phenotype was suppressed by the addition of mevalonate. HMG-CoA reductase activities of mutant MvaA proteins in the temperature sensitive mutants were lower than that of wild-type MvaA protein. Our findings from genetic and biochemical analyses suggest that mevalonate produced by HMG-CoA reductase is required for peptidoglycan synthesis for *S. aureus* cell growth.

In various organisms from bacteria to humans, HMG-CoA reductase is considered a rate-limiting enzyme for mevalonate synthesis. In higher organisms, mevalonate is a precursor for the synthesis of cholesterol and various types of isoprenoids^1^. The role of mevalonate in bacteria, however, has not yet been elucidated, because genetic studies of HMG-CoA reductase, which is responsible for the synthesis of mevalonate, are limited. *Escherichia coli* and *Bacillus subtilis*, which are widely used for genetic studies, do not have HMG-CoA reductase. In these bacteria, isopentenyl diphosphate (IPP), the starting molecule for the biosynthesis of various kinds of isoprenoids, is synthesized by the 2-*C*-methyl-D-erythritol-4-phosphate (MEP) pathway, which is independent of the mevalonate pathway^2^. On the other hand, several low G + C Gram positive-cocci like *S. aureus* do not have enzymes for the MEP pathway. Instead, they have enzymes, including HMG-CoA reductase, for the mevalonate pathway^3^,^4^,^5^. Therefore, it has been considered that *S. aureus* might produce IPP by the mevalonate pathway. Moreover, a deletion mutant of the *mvaA* gene coding HMG-CoA reductase in *S. aureus* showed cell growth depending on the addition of mevalonate to the culture media, suggesting that mevalonate synthesized by HMG-CoA reductase is necessary for *S. aureus* cell growth^3^. The essential molecules synthesized from mevalonate, however, have not yet been determined.

Bacterial temperature-sensitive mutants are extremely useful for studying the biological significance of enzymes that are coded by mutated genes. Furthermore, temperature-sensitive mutants are useful for identifying amino acid residues with critical roles in enzyme function. Our group previously identified the essential genes for *S. aureus* cell growth by isolating a number of temperature-sensitive mutants^6^,^7^,^8^,^9^,^10^,^11^,^12^,^13^,^14^. We reported *S. aureus* temperature-sensitive mutants of the *murB* or *murC* genes coding enzymes responsible for the synthesis of peptidoglycan^6^,^11^. We also reported amino acid residues of the enzymes essential for enzyme activity^8^,^13^. Temperature-sensitive mutants of the *mvaA* gene, however, have not yet been isolated or characterized in any bacteria, including *S. aureus*.

In the present study, we reveal the critical role of HMG-CoA reductase in the growth of *S. aureus* cells by using temperature-sensitive mutants of the *mvaA* gene. Our results demonstrated that this enzyme is required for peptidoglycan synthesis in *S. aureus*. Furthermore, we demonstrated that substituting isoleucine for methionine at the 77^th^ amino acid residue (M77I), valine for alanine at the 335^th^ amino acid residue (A335V), and tyrosine for cysteine at the 366^th^ amino acid residue (C366Y) of the *S. aureus* MvaA protein decreased the enzymatic activity.

## Results

### Isolation of *S. aureus* temperature-sensitive mutants of the *mvaA* gene

**Figure 1:**
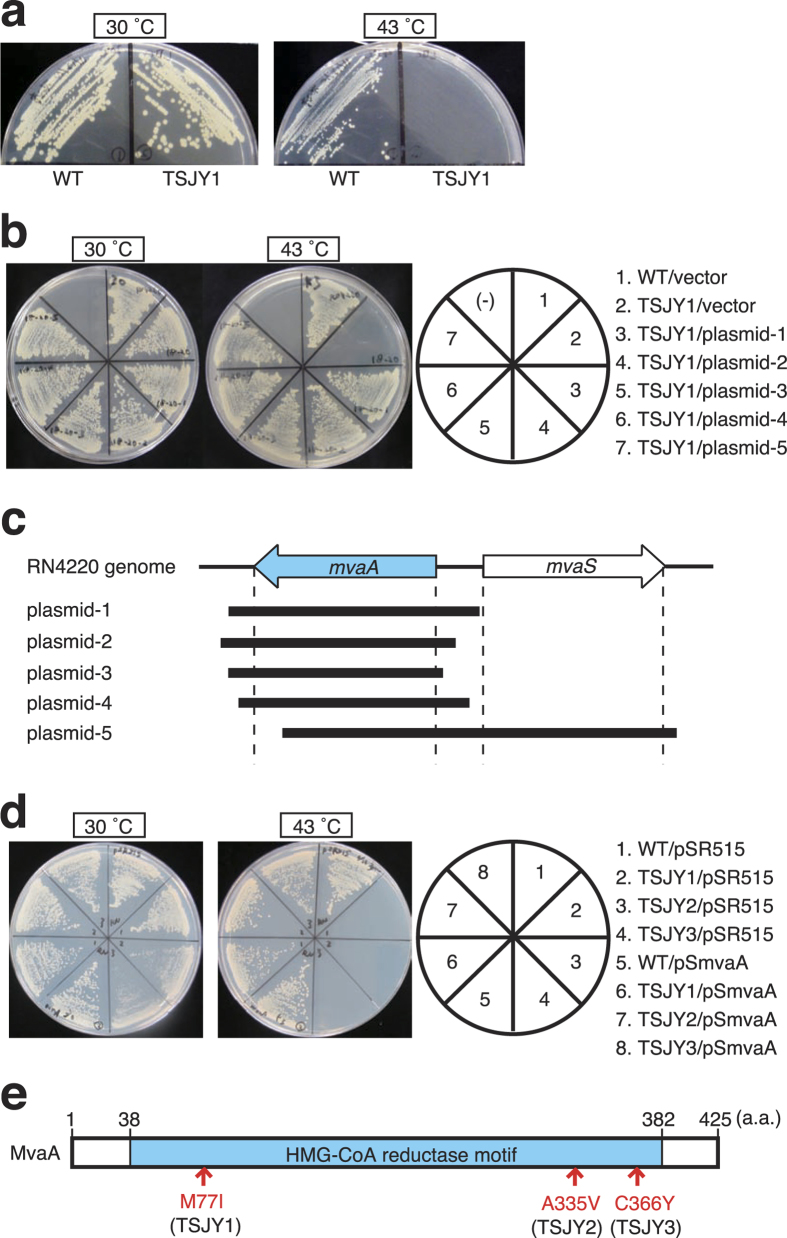
Isolation of temperature sensitive mutants of *mvaA* gene in *S. aureus*. (**a**) Overnight cultures of RN4220 (wild-type) and TSJY1 were diluted 100-fold and then streaked on LB0 agar plates. The plates were incubated at 30 °C or 43 °C for 24 h. (**b**) Isolation of DNA fragment from chromosomal DNA of wild-type cells complementing temperature-sensitive phenotype of TSJY1. Genome library of RN4220 was screened and transformants that could grow at 43 °C were isolated. Overnight cultures of RN4220 (wild-type) and the transformants were diluted 100-fold and then streaked on LB0 agar plates. The plates were incubated at 30 °C or 43 °C for 24 h. (**c**) Structure of genome DNA fragments that complemented the temperature-sensitive phenotype of TSJY1. (**d**) Complementation of temperature-sensitive phenotype of TSJY1, TSJY2, and TSJY3 by pSmvaA, a plasmid containing the open reading frame of the *mvaA* gene. Overnight cultures of RN4220 (wild-type)/pSR515, TSJY1/pSR515, TSJY2/pSR515, TSJY3/pSR515, RN4220/pSmvaA, TSJY1/pSmvaA, TSJY2/pSmvaA, and TSJY3/pSmvaA were diluted 500-fold and then streaked on LB0 agar plates. The plates were incubated at 30 °C or 43 °C for 24 h. (**e**) Arrows indicate the amino acid substitutions in the temperature sensitive-mutants TSJY1, TSJY2, and TSJY3.

We previously isolated the temperature-sensitive mutants in *S. aureus*^6^,^7^,^8^,^9^,^10^,^11^,^12^,^13^,^14^. In this study, we isolated *S. aureus* temperature-sensitive mutants of the *mvaA* gene coding HMG-CoA reductase. Overview of isolation method of temperature sensitive mutants in *S. aureus* is shown in Supplementary Fig. 1. TSJY1 (Fig. 1a), TSJY2, and TSJY3 were temperature-sensitive mutants obtained from *S. aureus* RN4220 strain treated with ethylmethanesulfonate. The temperature-sensitive phenotype of these three mutants was suppressed by introducing a plasmid containing the *S. aureus mvaA* gene coding HMG-CoA reductase (Fig. 1b–d). Sequence analysis revealed that the three mutants, TSJY1, TSJY2, and TSJY3, had single base substitutions, g231a, c1004t, and g1097a, in the *mvaA* gene, respectively (Supplementary Fig. 2). These mutations cause amino acid substitutions M77I, A335V, and C366Y, respectively (Fig. 1e). We then used phage transduction to verify whether the temperature-sensitive phenotype was due to mutations in the *mvaA* gene. As a representative example, the results with TSJY1 are described in Supplementary Fig. 3. The temperature-sensitive phenotype correlated with *mvaA* gene mutations of the TSJY1, TSJY2, and TSJY3 strains transferred by phage transduction (Table 1). Based on the results of the plasmid complementation analysis and the phage transduction experiment, we concluded that the temperature-sensitive phenotypes TSJY1, TSJY2, and TSJY3 each resulted from a mutation in the *mvaA* gene.

### Decreased peptidoglycan synthesis in *mvaA* gene mutants at a high temperature

**Figure 2:**
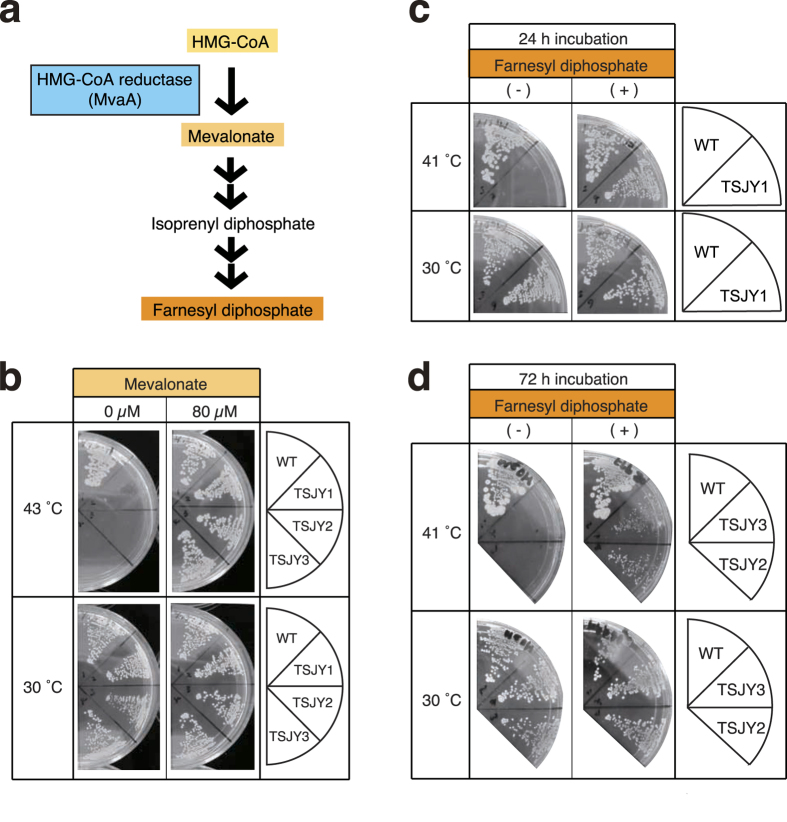
Suppression of temperature-sensitive phenotype in *mvaA* mutants by the addition of mevalonate or farnesyl diphosphate. (**a**) Illustration of the synthesis of farnesyl diphosphate from HMG-CoA. (**b**) Overnight culture of RN4220 (wild-type), TSJY1, TSJY2, and TSJY3 was diluted 500-fold and then streaked on LB0 agar plates with or without mevalonate (final concentration 80 μM). The plates were incubated at 30 °C or 43 °C for 24 h. (**c**) Overnight culture of RN4220 (wild-type) and TSJY1 was diluted 500-fold and then streaked on LB0 agar plates with or without farnesyl diphosphate (final concentration 80 μM). The plates were incubated at 30 °C for 2 h, and further incubated at 30 °C or 41 °C for 24 h. (**d**) Overnight culture of RN4220 (wild-type), TSJY2 and TSJY3 was diluted 500-fold and then streaked on LB0 agar plates with or without farnesyl diphosphate (final concentration 80 μM). The plates were incubated at 30 °C for 2 h, and further incubated at 30 °C or 41 °C for 72 h.

The MvaA protein, which has HMG-CoA reductase activity, is an enzyme that synthesizes mevalonate from HMG-CoA (Fig. 2a)^3^. We tested whether the temperature-sensitive phenotypes of the *mvaA* gene mutants TSJY1, TSJY2, and TSJY3, were suppressed by adding mevalonate to the culture medium. The results demonstrated that while TSJY1, TSJY2, and TSJY3 could not grow at 43 °C on LB0 agar plates, these *mvaA* gene mutants were able to grow at 43 °C in the presence of mevalonate (Fig. 2b). This means that the MvaA protein acts as a mevalonate synthetase in *S. aureus* cells and that its function is essential for *S. aureus* cell growth. We then examined whether mevalonate synthesized by MvaA protein has an essential role in *S. aureus* cell growth using *mvaA* gene mutants. IPP is a starting molecule for the biosynthesis of various kinds of isoprenoids in bacteria^2^. IPP is synthesized through either the mevalonate pathway or the MEP pathway, depending on the bacterial species. In *S. aureus,* which does not have genes coding for the enzymes of the MEP pathway, IPP is thought to be synthesized from mevalonate (Fig. 2a)^3^,^4^. In *E. coli*, IPP is changed to dimethylallyl diphosphate by IPP isomerase, and dimethylallyl diphosphate is subsequently changed to farnesyl diphosphate (FPP). FPP is a precursor of undecaprenyl phosphate (UP), which is a lipid carrier required for peptidoglycan synthesis^2^. Therefore, we hypothesized that IPP synthesized from mevalonate acts as a precursor of UP in *S. aureus*. Genetic studies of enzymes involved in peptidoglycan synthesis with various species of bacteria, including *S. aureus,* revealed that peptidoglycan synthesis is essential for bacterial growth^6^,^13^. We hypothesized that growth defect of the *mvaA* gene mutants at a high temperature are due to defective FPP synthesis, and thus evaluated this possibility. We examined whether the growth defect of the *mvaA* mutants at a high temperature would be suppressed by the addition of FPP to the agar medium. TSJY1, TSJY2, and TSJY3 were able to grow at 41 °C in the presence of FPP (Fig. 2c,d). The result suggests that FPP supplied from mevalonate is required for cell growth of *S. aureus*.

**Figure 3:**
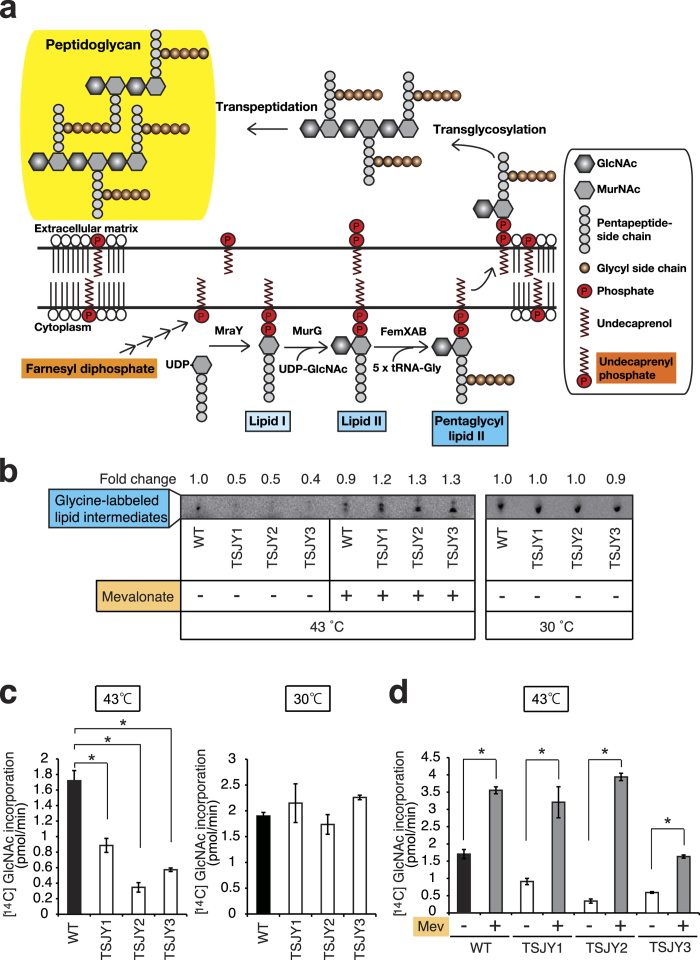
Decrease in the amount of lipid intermediates and peptidoglycan synthesis in *mvaA* mutants. (**a**) Illustration of the synthesis of peptideglycan from FPP. GlcNAc: β-1-4-*N*-acetylglucosamine, MurNAc: *N*-acetylmuramic acid. (**b**) Overnight cultures of RN4220 (wild-type), TSJY1, TSJY2, and TSJY3 were diluted 1000-fold, and further incubated at 30 °C or 43 °C for 5 h. Bacterial cells were disrupted and separated into a supernatant fraction and wall-membrane particulate fraction. The products of the *in vitro* reaction with [^14^C]glycine were separated by thin-layer chromatography as described in the Experimental Procedures. (**c**) Overnight cultures of RN4220 (wild-type), TSJY1, TSJY2, and TSJY3 were diluted 1000-fold, and further incubated at 30 °C or 43 °C. Cultures (OD_600_ = 0.1–0.5) were adjusted to OD_600_ = 0.5 in cell wall synthesis medium (CWSM) containing chloramphenicol (100 μg/ml), followed by incubation with [^14^C]* N*-acetylglucosamine at 30 °C or 43 °C for 30 min. Peptidoglycan synthesis was measured by [^14^C] *N*-acetylglucosamine incorporation. *P < 0.05. (**d**) Overnight cultures of RN4220 (wild-type), TSJY1, TSJY2, and TSJY3 were diluted 1000-fold, and further incubated with or without mevalonate (500 μM) at 30 °C or 43 °C. Cultures (OD_600_ = 0.1–0.5) were adjusted to OD_600_ = 0.5 in CWSM containing chloramphenicol (100 μg/ml), followed by incubation with [^14^C] *N*-acetylglucosamine with or without mevalonate (5 mM) in CWSM containing chloramphenicol (100 μg/ml) at 43 °C for 30 min. Peptidoglycan synthesis was measured by [^14^C] *N*-acetylglucosamine incorporation. Mev: mevalonate. *P < 0.05

UP produced from FPP supplies the short glycan chains for peptidoglycan synthesis in *S. aureus* (Fig. 3a)^15^. Pentaglycyl lipid II is formed from UP through lipid I and lipid II generation by several enzymatic reactions (Fig. 3a). We next determined the amounts of lipid intermediates, such as lipid I and lipid II, according to the present method using labeled glycine^16^,^17^. The amounts of glycine-labeled lipid intermediates in TSJY1, TSJY2, and TSJY3 grown at 43 °C were lower than that in wild-type (Fig. 3b). The phenotypes of the *mvaA* mutants were suppressed by mevalonate (Fig. 3b). These findings suggest that mevalonate is a precursor of the lipid intermediates that are required for peptidoglycan synthesis in *S. aureus*.

We then compared peptidoglycan synthesis between the *mvaA* mutants and wild-type cells. The rate of peptidoglycan synthesis was determined by the incorporation of radiolabeled *N*-acetylglucosamine^17^. The incorporation of radiolabeled *N*-acetylglucosamine was terminated in wild-type cells by treatment with vancomycin, an inhibitor of peptidoglycan synthesis, whereas the incorporation was not affected by norfloxacin, an inhibitor of DNA gyrase^18^. Therefore, we concluded that peptidoglycan synthesis in the cells could be accurately evaluated using this assay. We found that the incorporation of the radiolabeled *N*-acetylglucosamine in TSJY1, TSJY2, and TSJY3 at 43 °C was much lower than that of wild-type cells (Fig. 3c). We demonstrated that the protein synthesis rates determined by incorporation of [^35^S]methionine in TSJY1, TSJY2, and TSJY3 were indistinguishable from the wild-type after the temperature shift for at least 15 min (Supplementary Fig. 4). The addition of mevalonate led to an increase in the incorporation of the radiolabeled *N*-acetylglucosamine in each strain at 43 °C (Fig. 3d). Furthermore, adding FPP also increased the incorporation of the radiolabeled *N*-acetylglucosamine in TSJY1 at 43 °C (Supplementary Fig. 5). The increase of radiolabeled *N*-acetylglucosamine was not as significant with FPP than with mevalonate (Fig. 3d, Supplementary Fig. 5). These findings suggest that mevalonate is required for peptidoglycan synthesis in *S. aureus*.

**Figure 4:**
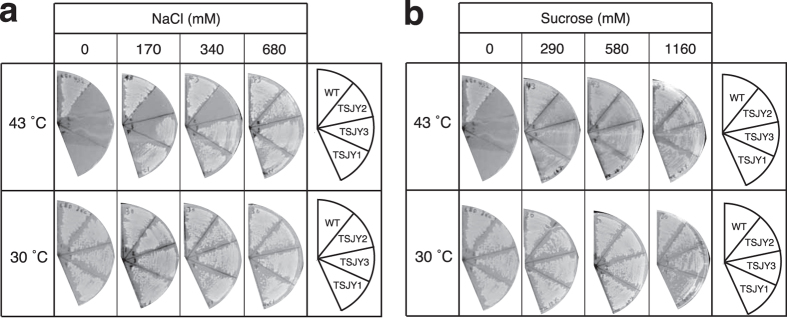
Suppression of temperature-sensitive phenotype in *mvaA* mutants under high osmotic conditions. (**a**,**b**) Overnight culture of RN4220 (wild-type), TSJY1, TSJY2, and TSJY3 was diluted 500-fold and then streaked on LB0 agar plates with or without NaCl (170, 340, or 680 mM) (**a**), or sucrose (290, 580, or 1160 mM) (**b**). The plates were incubated at 30 °C or 43 °C for 24 h.

Phenotypes caused by decreased peptidoglycan synthesis in *E. coli* and *S. aureus* can be suppressed by high osmotic conditions, such as a high concentration of NaCl or sucrose in the medium^6^,^19^,^20^. We previously reported that a temperature-sensitive phenotype of *S. aureus* strains with mutations in the *murC* gene coding the UDP-*N*-acetylmuramic acid:_L_-alanine ligase, which is essential for peptidoglycan synthesis, was suppressed by the addition of NaCl or sucrose to the culture medium^6^. It is thought that a decrease in peptidoglycan synthesis by bacteria under low osmotic conditions would disrupt the cytoplasmic membrane due to the osmotic difference between the inside and outside of the membrane. In contrast, cytoplasmic membranes of bacterial cells seem to be tolerant to high osmotic conditions^19^. In the present study, we examined whether the addition of NaCl or sucrose suppresses the temperature-sensitive phenotype of the *mvaA* mutants. Our findings indicated that TSJY1, TSJY2, and TSJY3 were able to grow at 43 °C in the presence of high concentrations of NaCl or sucrose (Fig. 4a,b). The results suggest that *mvaA* gene function in peptidoglycan synthesis is required for cell growth of *S. aureus*. In conclusion of these genetic studies, mevalonate plays a crucial role for peptidoglycan synthesis required for cell growth of *S. aureus*.

### Biochemical characterization of mutant MvaA proteins

**Figure 5:**
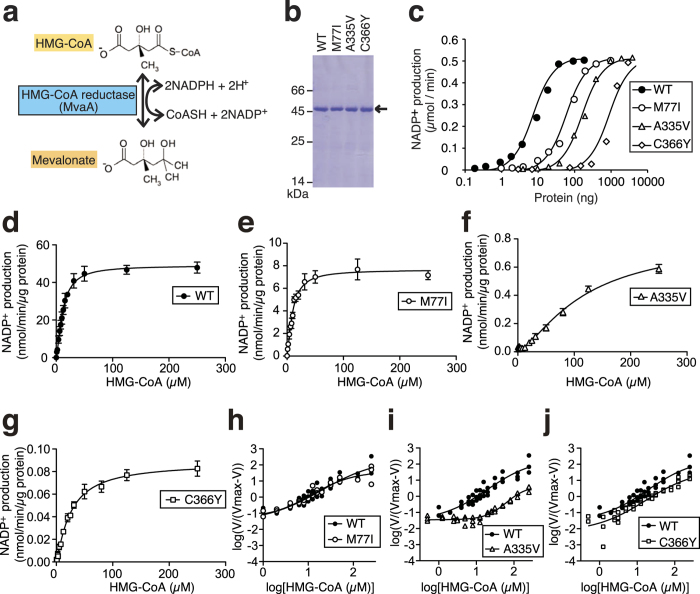
HMG-CoA reductase activities of purified MvaA proteins. (**a**) Enzymatic reaction to produce mevalonate from HMG-CoA mediated by MvaA protein. (**b**) Purified WT (wild-type), M77I, A335V, and C366Y mutant MvaA proteins were analyzed by SDS-PAGE. BSA (66 kDa), egg albumin (45 kDa), and α-chymotrypsinogen A (25 kDa) were used as molecular markers. (**c**) Titration of MvaA protein. Different concentrations of each MvaA protein were added to the reaction mixtures and incubated at 30 °C. (**d**–**g**) Wild-type, M77I, A335V, or C366Y mutant MvaA protein was added to a reaction mixture with different concentrations of HMG-CoA. The reaction was performed at 30 °C for 30 min. The amount of NADP^+^ produced in each sample was measured. (**h**–**j**) Hill plots for HMG-CoA of HMG-CoA reductase activity of wild-type, M77I, A335V, or C366Y mutant MvaA proteins.

We next performed a biochemical characterization to determine the molecular features of mutant MvaA proteins. First, we predicted the secondary structures of mutant MvaA proteins *in silico* to determine whether the mutations caused a dramatic structural failure. These mutations were not predicted to change the secondary structure of the MvaA protein (Supplementary Fig. 6). The MvaA protein is an HMG-CoA reductase, which catalyzes the production of both mevalonate from HMG-CoA and HMG-CoA from mevalonate reactions, which are naturally opposite (Fig. 5a).

We purified His-tag fused recombinant wild-type and mutant MvaA proteins from extracts of *E. coli* cells expressing these proteins at 16 °C. In the extract of *E. coli* that possesses control vector (Mock), activity of the HMG-CoA reductase did not be detected (Supplementary Table 3). We measured HMG-CoA reductase activity of wild-type MvaA protein in the soluble fractions (Fraction I) based on the amount of NADP^+^ produced (Supplementary Table 3). The soluble fraction of wild-type MvaA protein was further purified by nickel-affinity column chromatography. Wild-type MvaA protein was recovered by elution with a buffer containing imidazole (Fraction II). The specific activity of the final fraction of wild-type MvaA protein was 19-fold higher than that of Fraction I (Supplementary Table 3). Mutant MvaA proteins were purified in the same way. Each purified protein was detected as a single band on SDS-PAGE (Fig. 5b). The enzymatic reactions at 30 °C by the wild-type and M77I, A335V, and C366Y mutant MvaA proteins proceeded in a dose-dependent manner (Fig. 5c).

We then determined the kinetic parameters of enzymatic reactions catalyzed by wild-type and M77I, A335V, and C366Y mutant MvaA proteins. In the enzymatic reaction producing mevalonate from HMG-CoA and NADPH, the Vmax of the reaction catalyzed by M77I, A335V, and C366Y mutant MvaA proteins decreased to 1/6, 1/21, and 1/430 that of the wild-type MvaA protein, respectively (Fig. 5d–g, Supplementary Fig. 7a–d, and Table 2). These results suggest a decrease in HMG-CoA reductase activities of each MvaA mutant protein.

Rat liver microsomal HMG-CoA reductase is allosterically activated by NADPH^21^,^22^,^23^,^24^. We then determined Hill coefficient, which is a parameter for estimation of allosteric activation of an enzyme. The Hill coefficient of an enzyme allosterically activated by a substrate is higher than 1. The Hill coefficient of wild-type MvaA protein for HMG-CoA or NADPH measured by fitting with the Hill equation was 1.4 (Fig. 5h, Supplementary Fig. 7e, and Table 2). The Hill coefficient of the C366Y mutant MvaA protein for the HMG-CoA was lower than that of the wild-type, but the Hill coefficients of the M77I and A335V mutant MvaA proteins were similar to that of wild-type (Table 2). On the other hand, the Hill coefficients of the M77I and C366Y mutant MvaA proteins for NADPH were lower than that of the wild-type, but the Hill coefficient of the A335V mutant MvaA proteins was similar to that of the wild-type (Table 2). The *S*_0.5_ values, which are the substrate concentrations at half-maximal activity of A335V and C366Y mutant MvaA proteins for the HMG-CoA, were higher than that of the wild-type, but the *S*_0.5_ value of the M77I mutant MvaA protein was similar to that of the wild-type (Table 2). On the other hand, the *S*_0.5_ values of each mutant MvaA protein for NADPH were higher than that of the wild-type (Table 2). The results suggest allosteric activation of *S. aureus* MvaA protein by HMG-CoA and NADPH.

### Conservation of amino acid sequences and structural analyses of M77, A335, and C366 of MvaA in *S. aureus*

HMG-CoA reductase is conserved among a large number of organisms, such as humans, mice, nematodes, and yeast, and has an important role in the synthesis of biologically active substances^25^. Friesen and Rodwell reported the phylogenic tree of the HMG-CoA reductase and classified it as Class I and Class II based on the amino acid sequences^25^. We compared the amino acid sequences of HMG-CoA reductase among organisms and determined the evolutionary conservation of the M77, A335, and C366 of the *S. aureus* enzyme. The M77, A335, and C366 of the *S. aureus* enzyme were conserved in 66, 21, and 12 of 95 organisms having HMG-CoA reductase, respectively (Supplementary Fig. 8). The result suggests that M77 of the *S. aureus* enzyme is most conserved compared to the other two amino acids.

**Figure 6:**
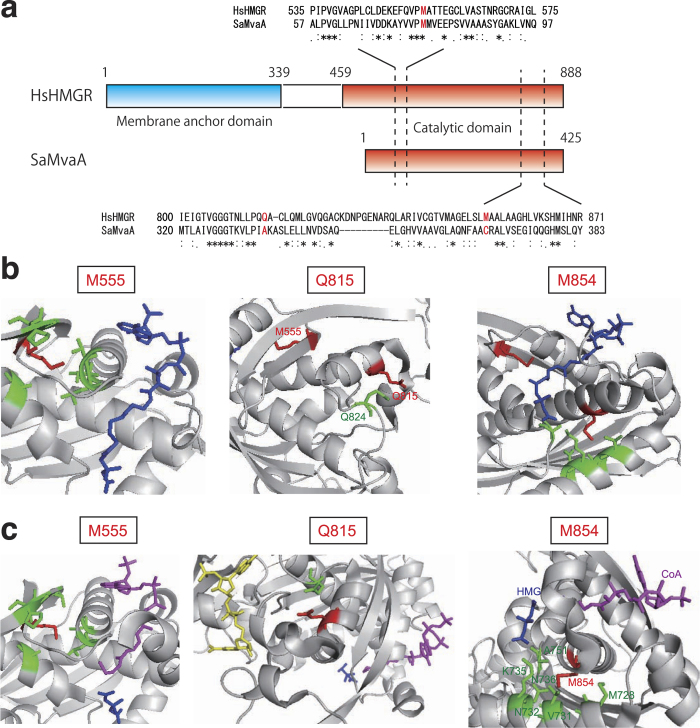
Location of the M555, Q815, and M854 in *Homo sapiens* HMG-CoA reductase. (**a**) Comparison of amino acid sequence in HMG-CoA reductase between *Homo sapiens* (HsHMGR) and *S. aureus* (SaMvaA). Residues corresponding to M77, A335, and C366 of *S. aureus* MvaA are indicated in red letters. (**b**) A complex of *Homo sapiens* HMG-CoA reductase and HMG-CoA (PDB ID: 1DQ9)^27^. Red: the M555, Q815, or M854; Green: amino acid residues locating within 4Å from the residues indicated in red; Blue: HMG-CoA. (**c**) A complex of *Homo sapiens* HMG-CoA reductase and HMG, CoA, NADP^+^ (PDB ID: 1DQA)^27^. Red: the M555, Q815, or M854; Green: amino acid residues locating within 4Å from the residues indicated in red; Blue: HMG, Yellow: NADP^+^, Purple: CoA.

Recognition sites of substrates in human HMG-CoA reductase were revealed by co-crystal structures of the enzyme and substrates^26^,^27^. We tested whether the M77, A335, and C366 of the HMG-CoA reductase in *S. aureus* exist around the recognition sites of the substrates based on the structural analyses of human HMG-CoA reductase. The alignment analysis of amino acid residues showed that the M77, A335, and C366 of the HMG-CoA reductase in *S. aureus* correspond to the M555, Q815, and M854 of the human HMG-CoA reductase, respectively (Fig. 6a). In the case of the HMG-CoA reductase bound for HMG-CoA, the M555, Q815, and M854 of the human HMG-CoA reductase locate a little far from the binding sites of HMG-CoA and mevalonate, the substrate and product of the enzyme (Fig. 6b). In the case of the HMG-CoA reductase bound for HMG, CoA, and NADP^+^, the M555, Q815, and M854 were not located within 4Å from the substrates (Fig. 6c). These three amino acid residues might not be able to bind the substrates HMG-CoA, methionine, HMG, and CoA.

## Discussion

In the present study, we isolated and characterized three temperature-sensitive mutants of the *mvaA* gene in *S. aureus* to reveal an essential role of HMG-CoA reductase for cell growth. The temperature-sensitive phenotype of TSJY1, TSJY2, and TSJY3, with mutations in the *mvaA* gene, was verified by plasmid complementation analyses and phage transduction experiments. *E. coli* and *B. subtilis*, which are frequently used for genetic studies, do not have the *mvaA* gene. To our knowledge, this is the first report describing the isolation of temperature-sensitive mutants of the *mvaA* gene in bacteria.

The temperature-sensitive phenotype in the *mvaA* gene mutants was suppressed by supplementation with mevalonate and FPP. These findings suggest that mevalonate is a source of FPP. Studies of peptidoglycan synthesis in *E. coli* and *B. subtilis* revealed that FPP is a source of UP, a lipid carrier that supplies the glycan chains for peptidoglycan synthesis^28^. The decreased peptidoglycan synthesis in the *mvaA* gene mutants might be due to insufficient amounts of FPP in the cells. The growth defects caused by the decreased peptidoglycan synthesis in *E. coli* and *S. aureus* are suppressed by high osmotic conditions^6^,^19^,^20^. Thus, suppression of the temperature-sensitive phenotype in the *mvaA* gene mutants by high osmotic conditions can be explained by decreased peptidoglycan synthesis in the mutants. Transcriptome analyses of *S. aureus* genes in which the expression levels were altered by treatment with cell wall synthesis-inhibitors, such as oxacillin, D-cycloserine, bacitracin^29^, and vancomycin^30^,^31^,^32^ were performed. Fifteen genes, whose expression was increased, and two genes, whose expression was decreased by oxacillin, D-cycloserine, bacitracin, and vancomycin, are proposed to be cell wall stimulons involved in cell wall synthesis. Moreover, Balibar *et al.* reported that decreased expression of the *mvaA* gene using an IPTG-regulated system led to altered expression of the cell wall stimulons^33^. This finding suggests that *mvaA* gene in *S. aureus* contributes to peptidoglycan synthesis. The present study extended these findings using temperature-sensitive mutants of the *mvaA* gene.

The HMG-CoA reductase activity of purified *S. aureus* MvaA protein was allosterically enhanced by HMG-CoA. Rat liver microsomal HMG-CoA reductase is allosterically activated by NADPH^21^,^22^,^23^,^24^. To our knowledge, this is the first report that HMG-CoA reductase is activated allosterically by HMG-CoA. Multimeric formation of protein would affect Hill coefficient calculations with regard to the number of binding sites. The *Pseudomonas mevalonii* MvaA protein forms dimers^34^. We demonstrated that *S. aureus* MvaA protein also forms dimers by gel filtration analysis (Supplementary Fig. 9). Therefore, we assumed that the MvaA protein in *S. aureus* recognizes the substrates in a cooperative manner by forming a dimer.

In M77I mutant MvaA protein, the Hill coefficient and the *S*_0.5_ value for HMG-CoA were not changed, but the Hill coefficient for NADPH was lower than that of the wild-type and the *S*_0.5_ value for NADPH was higher than that of the wild-type. The results suggest that the M77I mutation in MvaA protein causes a defect in the allosteric activation by NADPH. The decreased activity by M77I mutation in MvaA protein seems to decreasing affinity for NADPH by its reducing allosteric activation. Moreover, we performed *in silico* structural analysis and indicated that the M77I mutation in MvaA protein does not affect the position of the helix that recognizes HMG-CoA (Supplementary Fig. 10). In the A335V mutant MvaA protein, the Hill coefficients for HMG-CoA and NADPH were not altered, but the *S*_0.5_ values were lower than that of the wild-type. This finding suggests that the A335V mutation in MvaA protein causes defects in the substrate recognition of HMG-CoA and NADPH. The decreased activity of the A335V mutation in the MvaA protein seems to be due to a decreased affinity for HMG-CoA and NADPH without a concomitant decrease in their allosteric activations. In the C366Y mutant MvaA protein, the Hill coefficients for HMG-CoA and NADPH were lower than that of the wild-type, but the *S*_0.5_ values were higher than that of the wild-type. The results suggest that the C366Y mutation in MvaA protein causes defects not only in the allosteric activation by HMG-CoA and NADPH, but also in the recognition of these substrates. The decreased activity of the C366Y mutation in MvaA protein seems to be due to the decreased affinity for HMG-CoA and NADPH with a concomitant decrease in their allosteric activation.

The M77 residue of the *S. aureus* enzyme is conserved in 66 of 95 organisms having HMG-CoA reductase. In eukaryotic Class I enzymes, the methionine residue is conserved in 45 organisms, except in Cockroach, Yew, and *Schistosoma mansoni*. On the other hand, in case of Archaea and Eubacteria Class I enzymes, the corresponding amino acid residue is leucine in 19 of 21 organisms, the exceptions being *S. solfataricus*, and *Halobacterium sp.* It seems that the methionine residue of Class I enzymes changed to a leucine residue from a methionine residue at an evolutionary branch of the Archaea and Eubacteria. We assume that the methionine residue of *S. solfataricus* and *Halobacterium sp.* has a reverse mutation. In the case of Class II enzymes, the methionine residue is conserved in 19 of 26 organisms, the exceptions being *S. mutans*, *S. agalactiae*, *S. pyogenes*, *S. pneumoniae*, *P. mevalonii*, *C. aurantiacus*, and *B. burgdorferi*. Therefore, the M77 residue of the HMG-CoA reductase in *S. aureus* is highly conserved among eukaryotes. We assume that HMG-CoA reductases, which have the conserved methionine residue, may be activated allosterically by NADPH. The A335 residue of the *S. aureus* enzyme is well conserved in Class II. On the other hand, in case of eukaryotic Class I enzymes, the alanine residue is conserved in 4 of 48 organisms, and, in case of Archaea and Eubacteria Class I enzymes, the alanine residue is not conserved. The alanine residue is conserved in *Listeria* species, but not in *Streptococcus* species. We assume that the alanine residue was varied to valine in divergence of *Listeria* species and *Streptococcus* species. The C366 residue of the *S. aureus* enzyme is conserved in *Staphylococcus* species and some vegetable species. On the other hand, the cysteine residue is not conserved in *Lactococcus lactis*, *Enterococcus faecium*, and *Lactobaccilus plantarum*. In divergence of these species and *Staphylococcus* species, the cysteine residue of MvaA protein in *Staphylococcus* species might be generated by a mutation.

From the results based on the crystal structure of human HMG-CoA reductase, we assumed that the M77, A335, and C366 residues of the HMG-CoA reductase in *S. aureus* do not directly bind the substrates. Therefore, we identified amino acid residues essential for the HMG-CoA reductase activity from analysis of temperature-sensitive mutants, and these amino acid residues are difficult to predict from analysis of crystal structure. From the biochemical and structural analyses, these amino acid residues of the enzyme might play an important role in the step of structural change by substrate recognition.

**Figure 7:**
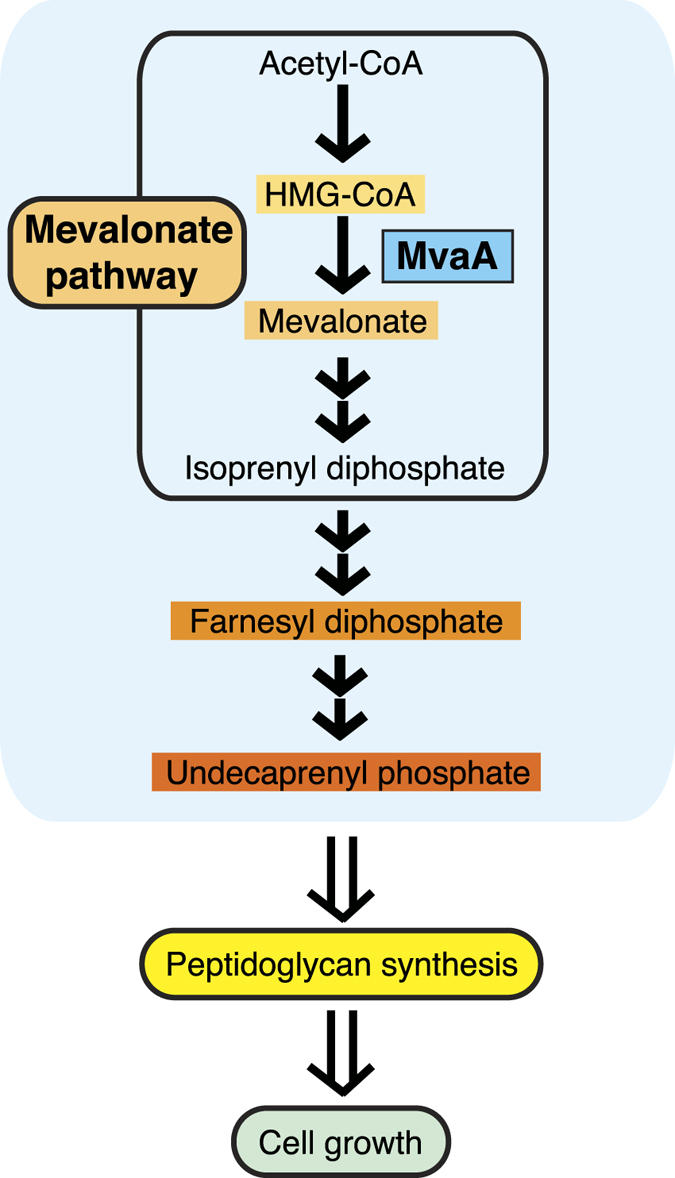
Schematic drawing demonstrating that mevalonate acts as a source of undecaprenyl phosphate and contributes to peptidoglycan synthesis for cell growth in *S. aureus*.

In conclusion, mevalonate produced by HMG-CoA reductase plays a crucial role for peptidoglycan synthesis for *S. aureus* cell growth (Fig. 7). Multidrug-resistant *S. aureus* is a serious clinical problem. Peptidoglycan synthesis inhibitors such as penicillin and vancomycin are currently used to treat *S. aureus* infections, but strains resistant to these drugs have recently been isolated all over the world^35^,^36^. Therefore, novel target proteins for the development of new inhibitors against peptidoglycan synthesis are desired^35^. Our present study suggests that MvaA protein is required for the synthesis of peptidoglycans in *S. aureus*. Farnesol inhibits HMG-CoA reductase activity and *S. aureus* cell growth^37^. We demonstrated that mevalonate suppressed the farnesol-induced inhibition of peptidoglycan synthesis in *S. aureus* (Supplementary Fig. 11). This finding indicates that drug inhibition of bacterial HMG-CoA reductase causes a decrease in the concentration of mevalonate in the cytoplasm, resulting in the inhibition of peptidoglycan synthesis. Thus, the MvaA protein is a promising target for the development of antibiotics to treat human patients infected with *S. aureus*.

## Methods

### Bacterial strains and culture media

The *S. aureus* and *E. coli* strains used in this study are listed in Supplemental Table 1. *E. coli* JM109 (Takara Bio) was used as the host for plasmid construction. *E. coli* BL21 (DE3)/pLysS (Novagen) was used to produce recombinant MvaA proteins. *E. coli* and *S. aureus* cells were grown in Luria-Bertani (LB) 10 medium [1% (w/v) bactotryptone (Becton Dickinson), 0.5% (w/v) yeast extract (Becton Dickinson), and 1% (w/v) NaCl], or Tryptic Soy Broth (TSB) medium (Becton Dickinson) with 12.5 μg/ml chloramphenicol as needed.

### Examination of temperature sensitivity of *S. aureus*

*S. aureus* cells were grown in LB10 medium at 30 °C. Overnight cultures were diluted 100-fold with 0.9% NaCl and streaked on LB0 (1% [w/v] bactotryptone [Becton Dickinson], 0.5% [w/v] yeast extract [Becton Dickinson] without NaCl) agar plates. The plates were incubated at permissive (30 °C) or non-permissive (41–43 °C) temperatures for 1 to 5 days and colonies appeared. Suppression experiments of the temperature-sensitive phenotype by the addition of chemicals were performed by adding (±)-mevalonolactone (Sigma), which turns to mevalonate in water, or farnesyl pyrophosphate (Sigma) to LB0 agar. High osmotic conditions were produced by adding NaCl or sucrose to the agar medium as previously described^38^.

### Isolation of *mvaA* temperature-sensitive mutants

Temperature-sensitive mutants were obtained from RN4220^39^. For mutagenesis, overnight cultures of RN4220 were diluted 100-fold with medium, followed by culture with 0.2% ethylmethanesulfonate (Sigma) at 30 °C overnight as described previously^14^. For gene transformation, competent *S. aureus* cells were prepared as described previously^14^. Briefly, *S. aureus* cells in the early logarithmic growth phase (OD _600_ = 0.3) in LB10 medium at 30 °C were collected and suspended in 500 mM sucrose solution. *S. aureus* cells were washed twice with 500 mM sucrose. Transformation of *S. aureus* with plasmids was performed as described previously^14^. To identify the genes that complement the temperature-sensitive phenotype, a library of *S. aureus* genomic DNA, which was a mixture of plasmids carrying DNaseI-digested 2- to 4-kb fragments of *S. aureus* RN4220 chromosomal DNA in the *Sma*I site of a shuttle vector, pSR515, was constructed and electroporated into mutant competent cells. Transformant colonies that grew at 43 °C on LB0 agar plates containing 12.5 μg/ml chloramphenicol were isolated. Plasmid DNA was extracted from the transformants^14^, and the nucleotide sequences inserted in the *Sma*I site of the plasmids were determined using primers P3 and P4, and compared with the genomic DNA sequence of *S. aureus* N315^40^. Mutations of the *mvaA* gene of the temperature-sensitive mutants were determined by sequencing analysis using the primers mvaAseq1F, mvaAseq1R, mvaAseq2F, and mvaAseq2R. We performed the sequencing analyses at least two times to confirm the point mutations in the *mvaA* gene. Details of the plasmids and primers used in this study are shown in Supplemental Table 1 and 2, respectively. The plasmid pS*mvaA* carries the *S. aureus mvaA* gene region, which was amplified with PCR using RN4220 chromosomal DNA as a template, using primers mvaAup255 and mvaAend, and inserted in the *Sma*I site of pSR515.

### Phage transduction of *S. aureus*

The downstream region of the *mvaS* gene was amplified by PCR from RN4220 chromosomal DNA using primers Fside-mvaA and Rside-mvaA, which were added to the *Kpn*I and *Xba*I sites at the ends of the amplified fragment, respectively, and inserted in the *Kpn*I and *Xba*I site of pCK20 resulting in the plasmid pCK20mvaA-side. The plasmid was delivered into the TSJY1, a *mvaA* gene point mutant, competent cells by electroporation and transformant colonies resistant to chloramphenicol were isolated on LB10 agar plates containing 12.5 μg/ml chloramphenicol. The transformant was used as a donor for phage transduction, which was integrated with the chloramphenicol-resistant gene into the downstream region of the *mvaS* gene by homologous recombination events (Supplementary Fig. 3). Phage transduction was performed as described previously^41^. Phage transductant colonies resistant to chloramphenicol were isolated. RN4220 introducing the chloramphenicol-resistant gene was used as a donor to integrate the chloramphenicol-resistant gene into other mutants.

### Construction of wild-type and mutant MvaA protein expression plasmids

The *mvaA* gene was amplified with PCR from *S. aureus* RN4220, TSJY1, TSJY2, and TSJY3 chromosomal DNA using primers FmvaA-His and RmvaA-His, which introduced *Xho*I and *Bam*HI sites at the ends of the amplified fragment. The products were digested with *Xho*I and *Bam*HI and then respectively ligated into the *Xho*I and *Bam*HI sites of pET28a (Novagen) in-frame with the N-terminal histidine tag, resulting in plasmids pHis-mvaA, pHis-mvaA-M77I, pHis-mvaA-A335V, and pHis-mvaA-C366Y. To confirm that no errors had been introduced, inserts were sequenced using the primer T7 promoter and T7 terminator.

### Purification of *S. aureus* wild-type and mutant MvaA proteins

BL21(DE3)pLysS (Novagen) were transformed with plasmids of the pHis-mvaA series. Each strain was grown in 1 L of LB 10 medium at 37 °C for 2 h and 16 °C for 1 h, after which the culture was adjusted to 0.5 mM isopropyl 1-thio-β -D- galactopyranoside (IPTG), and protein expression was induced at 16 °C for 3 h. Bacterial cells were harvested by centrifugation, resuspended in lysis buffer (50 mM NaH_2_PO_4_, 500 mM NaCl, 1 mg/ml lysozyme), frozen in liquid N_2_, thawed, and sonicated 2 times for 30 s. Cellular debris was removed by centrifugation at 10,000 rpm for 10 min. The cleared cell lysates were mixed with a nickel-chelating resin (ProBond™ Resin, Invitrogen) and incubated at 4 °C for 1 h. The resin was then packed into a column and washed with a washing buffer (50 mM NaH_2_PO_4_, 500 mM NaCl, and 10 mM or 50 mM imidazole). The recombinant proteins were recovered with an elution buffer (50 mM NaH_2_PO_4_, 500 mM NaCl, 340 mM imidazole). The eluted fractions were dialyzed with dialysis buffer (50 mM Tris/HCl [pH7.5], 150 mM NaCl, 20% glycerol) for 24 h. Each fraction was analysed by SDS-polyacrylamide gel electrophoresis (SDS-PAGE), and proteins were stained with Coomassie Brilliant Blue. Protein concentrations were determined by the Bradford method using bovine serum albumin (BSA) as a standard. The proteins were frozen in liquid N_2_ and stored at −80 °C.

### HMG-CoA reductase activity assay

Spectrophotometric assays of HMG-CoA reductase activity of the oxidation or reduction of NADPH was performed using a DU730 spectrophotometer (Beckman Coulter) at 340 nm as described previously^3^. One hundred microliters of reaction medium with 5% glycerol and 100 μg/ml BSA were incubated at 30 °C in a water bath. Assay conditions for reductive deacylation of HMG-CoA to mevalonate and oxidative acylation of mevalonate to HMG-CoA were as described previously^3^. Curve fittings by non-linear regression for kinetic parameters were generated by Hill equations using Prism (GraphPad Software, Inc., La Jolla, CA).

### Measurement of peptidoglycan synthesis in *S. aureus*

Incorporation of *N*-acetyl-D-[^14^C]glucosamine into acid-insoluble fractions was monitored as described previously^17^. Cells from overnight cultures of RN4220 and TSJY1 grown in LB 10 medium were diluted in 1 ml LB0 medium, followed by incubation at 30 °C or 43 °C. After 5 h, the incubated cultures were adjusted to OD_600_ = 1.0 in LB0, followed by incubation at 30 °C or 43 °C for 15 and 30 min, respectively. An aliquot (200 μl) was sampled, and an equal volume of 10% trichloroacetic acid was added to a final concentration of 5%, followed by filtration through nitrocellulose membrane filters (HA, 0.45 μm; Millipore). Radioactivity of acid-insoluble fractions retained on the filters was measured in a liquid scintillation counter (Beckman).

### Preparation of wall-membrane particulate and cytosolic (supernatant) fraction of *S. aureus*

Wall-membrane particulate and cytosolic (supernatant) fractions were prepared as previously described^17^ with minor modifications. To obtain the wall-membrane particulate of *S. aureus* RN4220 and each *mvaA* temperature-sensitive mutant, overnight cultures of each strain were diluted in LB0 medium with or without 500 μM mevalonate and further cultivated at 30 °C or 43  °C with shaking for 5 h. Cultured cells were harvested and washed with 50 mM Tris-HCl buffer (pH 8.0) containing 0.1 mM MgCl_2_ (buffer A) and disrupted 5 times with a high-pressure homogenizer Emulsi Flex-B15 (AVASTIN) at 39,000 psi. Disrupted cells were centrifuged at 8000 g for 10 min and each precipitated wall-membrane particulate was resuspended in buffer A. For preparation of the cytosolic fraction, RN4220 was grown, harvested, and disrupted as described above. The supernatant of disrupted cells after centrifugation was collected and dialyzed in buffer A containing 100 mM NaCl. The wall-membrane particulate and the supernatant of each strain were frozen in liquid nitrogen and stored at −80 °C.

### Detection of lipid intermediates

Enzymatically synthesized lipid intermediates were detected with labeled glycine as described previously^17^. The reaction mixture contained 60 mM Tris-HCl (pH 8.5), 30 mM MgCl_2_, 1 mM 2-mercaptoethanol, 330 μM UDP-GlcNAc, 1.7 mM ATP, 12.5 μg protein/250 μL of the RN4220 supernatant, 9.25 kBq/sample [^14^C]glycine (PerkinElmer), and 37.5 μg/250 μL of wall-membrane particulate fraction. The reaction was performed at 30 °C for 24 h. Labeled lipids were extracted from the reaction mixture using the Bligh-Dyer method and the dried lipid fraction was resuspended in chloroform. The lipid extract was developed on thin-layer chromatography silica gel 60 (Millipore) with chloroform/methanol/water/ammonia (88:48:11:1, v/v/v/v)^42^ as the solvent for 1.5 h. The radioactive spots on the plate were detected after exposure to an imaging plate for 10 days and scanning with a Typhoon FLA 9000 (GE Healthcare).

### Phylogenic analysis of amino acid residue M77, A335, and C366 in *S. aureus* MvaA protein

A phylogenetic tree of HMG-CoA reductases was reported by Friesen and Rodwell^25^. GenBank accession numbers of HMG-CoA reductases were obtained from Table 1 of the report. The alignment of the primary structure was generated using ClustalW^43^. Amino acid residues corresponding to the amino acid residue M77, A335, and C366 in *S. aureus* MvaA protein were identified from the alignments.

### Structure of HMG-CoA reductases

Three-dimensional structure of Homo sapiens HMG-CoA reductase was obtained from the Protein Data Bank (PDB, http://www.rcsb.org/pdb/home/home.do); PDB ID of *Homo sapiens* HMG-CoA reductase: 1DQA and 1DQ9)^27^. Structures were denoted using PyMOL (http://www.pymol.org) represented in cartoon shape. The focused amino acid residues and substrates were represented in stick shape.

### Statistical analysis

Data are shown as means ± standard deviation (SD). Statistical significance between groups was evaluated using a two-tailed Student’s *t* test. A p-value of less than 0.05 was considered statistically significant.

## Additional Information

**How to cite this article**: Matsumoto, Y. *et al.* A critical role of mevalonate for peptidoglycan synthesis in *Staphylococcus aureus. Sci. Rep.* **6**, 22894; doi: 10.1038/srep22894 (2016).

## Supplementary Material

Supplementary Information

## Figures and Tables

**Table 1:** Summary of phage transduction analyses.

Genotype^a^	Phenotype^b^	
	Temperature-resistant	Temperature-sensitive
Donor; TSJY1 (Cm^r^), Recipient; RN4220		
*mvaA* WT	13	0
*mvaA* g231a	0	66
Donor; RN4220 (Cm^r^), Recipient; TSJY1		
*mvaA* WT	108	0
*mvaA* g231a	0	10
Donor; TSJY2 (Cm^r^), Recipient; RN4220		
*mvaA* WT	15	0
*mvaA* c1004t	0	92
Donor; RN4220 (Cm^r^), Recipient; TSJY2		
*mvaA* WT	95	0
*mvaA* c1004t	0	14
Donor; TSJY3 (Cm^r^), Recipient; RN4220		
*mvaA* WT	21	0
*mvaA* g1097a	0	84
Donor; RN4220 (Cm^r^), Recipient; TSJY3		
*mvaA* WT	88	0
*mvaA* g1097a	0	16

Genotype and phenotype were determined about 622 transductants, which show chloramphenicol resistance.

^a^Genotype was determined by sequence analysis.

^b^Phenotype was determined with or without colony formation on LB0 agar plate at 43 °C.

**Table 2:** Kinetic parameters of *S. aureus* MvaA proteins.

MvaA protein	*V*_max_ (nmol/min/μg)	*S*_*0.5*_		*n*_*H*_	
		HMG-CoA (μM)	NADPH (μM)	HMG-CoA (μM)	NADPH (μM)
Wild-type	43 ± 1	11 ± 4	11 ± 1	1.4 ± 0.1	1.4 ± 0.1
M77I	7 ± 1^*c*^	10 ± 1	29 ± 8^*a*^	1.4 ± 0.2	1.1 ± 0.1^*a*^
A335V	2 ± 1^*c*^	120 ± 24^*b*^	240 ± 120^*a*^	1.5 ± 0.3	1.3 ± 0.2
C366Y	0.1 ± 0^*c*^	26 ± 3^*b*^	34 ± 10^*a*^	1.2 ± 0.1^*a*^	1.0 ± 0.1^*a*^

By fitting with the Hill equation, the parameters were determined with *S*_*0.5*_ as the substrate concentration at half-maximal activity and *n*_*H*_ as the Hill coefficient. The values are means ± S.D. of three independent experiments. ^*a*^*p* < 0.05 (vs Wild-type). ^*b*^*p* < 0.01 (vs Wild-type). ^*c*^*p* < 0.000001 (vs Wild-type).
