# Supplementary material for: A critical role of mevalonate for peptidoglycan synthesis in Staphylococcus aureus
Source: Sci Rep. 2016 Mar 10;6:22894. doi: 10.1038/srep22894 (PMC4790635; doi:10.1038/srep22894)
Supplement: Supplementary Information [file srep22894-s1.pdf]

Supplementary Information

A critical role of mevalonate for peptidoglycan synthesis in *Staphylococcus aureus*

Yasuhiko Matsumoto [1,2], Jyunichiro Yasukawa [1,2], Masaki Ishii [1,2], Yohei Hayashi [2], Shinya Miyazaki [2], and Kazuhisa Sekimizu[2]

[1]These authors contributed equally to this work.
[2]Laboratory of Microbiology, Graduate School of Pharmaceutical Sciences, The University of Tokyo

Supplemental Table 1 List of bacterial strains and plasmids used in this study

Strains or plasmids	Genotypes or characteristics	Sources or references
Strains		
E.coli		
JM109	General purpose host strain for cloning	Takara Bio
BL21(DE3)pLysS	General purpose host strain for expression of recombinant proteins	Takara Bio
S.aureus		
RN4220	NCTC8325-4, restriction mutant	[1]
TSJY1	RN4220 *mvaA* (M77I)	This study
TSJY2	RN4220 *mvaA* (A335V)	This study
TSJY3	RN4220 *mvaA* (C366Y)	This study
Plasmids		
pET-28a	T7 promoter based expression vector , Km^r	Novagen
pHis-MvaA	pET-28a with His-tagged mutated *mvaA* (Wt)	This study
pHis-MvaA-M77I	pET-28a with His-tagged mutated *mvaA* (M77I)	This study
pHis-MvaA-A335V	pET-28a with His-tagged mutated *mvaA* (A335V)	This study
pHis-MvaA-C366Y	pET-28a with His-tagged mutated *mvaA* (C366Y)	This study
pSR515	*E. coli- S. aureus* shuttle vector ; Cm^r	[2]
pSmvaA	pSR515 with intact *mvaA* from RN4220	This study
pCK20	*S. aureus* integration vector ; Cm^r	[3]
pCKmvaA-side	pCK20 with a genome region near by *mvaA*	This study

Supplemental Table 2 PCR primers used in this study.

Target	Primer	Sequence(5'-3')
mvaA for complementation	mvaAup25	AAGGTTTTACATCCTTTCAAATTTATAAAAG
	mvaAend	CTATTGTTGTCTAATTTCTTGTAAAATGC
mvaA for sequence	mvaAseq1F	CGAATATCATTGTGGACGATAAG
	mvaAseq1R	CTTATCGTCCACAATGATATTCG
	mvaAseq2F	TATCGTGGTATTGCAACATGG
	mvaAseq2R	CCATGTTGCAATACCACGATA
mvaA for phage transduction	Fside-mvaA	CGCGGTACCAATTCAGAAATTCGTTTTTCA
	Rside-mvaA	GCGTCTAGAGCCTATTTGACACATTATTGA
MvaA for over production	FMvaA-His	CGCGGATCCATGCAAAGTTTAGATAAGAAT
	RMvaA-His	GCGCTCGAGCTATTGTTGTCTAATTTCTTG
Insert region in genome library	p3	GCCTCGCAGAGCATACGG
	p4	GCCTCGCAGAGCATACGG

Supplemental Table 3 Purification table for wild-type *S. aureus* MvaA protein

[a] 1 unit = 1 µmol of NADPH oxidized/min

Fraction	Purification step	Total protein (mg)	Total activity[a] (U)	Yield (%)	Specific activity[a] (U/mg)	Fold
**Wild-type**						
I	Cell lysate	42.4	316	100	7.5 ± 0.2	1
II	Nickel affinity column	6.5	933	300	143 ± 1	19
**Mock**						
I	Cell lysate	45.6	0	-	0	-
II	Nickel affinity column	3.2	0	-	0	-

Supplemental Figures

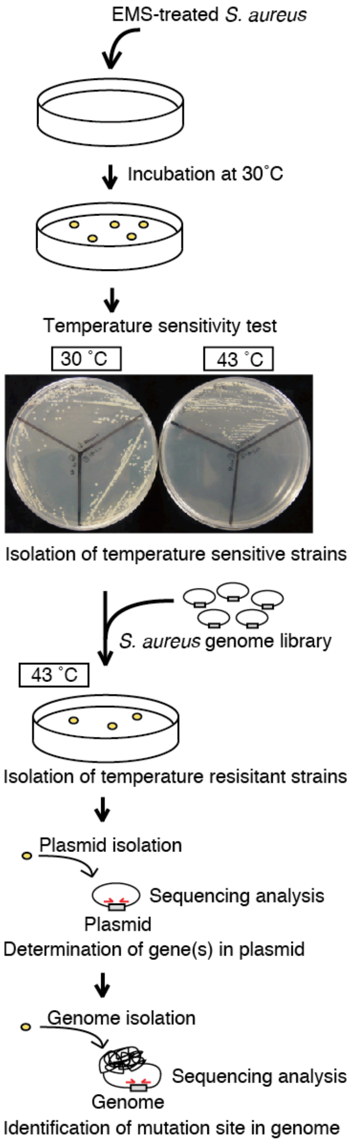

Supplementary Figure 1. Overview of isolation method of temperature sensitive mutants in *S. aureus*.
EMS: ethylmethanesulfonate.

```
RN4220   201 TGTGGACGATAAGGCATATGTTGTACCTATGATGGTGGAAGAGCCTTCAG  250
TSJY1    201 TGTGGACGATAAGGCATATGTTGTACCTATAATGGTGGAAGAGCCTTCAG  250
TSJY2    201 TGTGGACGATAAGGCATATGTTGTACCTATGATGGTGGAAGAGCCTTCAG  250
TSJY3    201 TGTGGACGATAAGGCATATGTTGTACCTATGATGGTGGAAGAGCCTTCAG  250

RN4220   971 TCGTTGGCGGTGGTACAAAAGTATTACCAATTGCTAAAGCTTCATTAGAG 1020
TSJY1    971 TCGTTGGCGGTGGTACAAAAGTATTACCAATTGCTAAAGCTTCATTAGAG 1020
TSJY2    971 TCGTTGGCGGTGGTACAAAAGTATTACCAATTGTTAAAGTTTCATTAGAG 1020
TSJY3    971 TCGTTGGCGGTGGTACAAAAGTATTACCAATTGCTAAAGCTTCATTAGAG 1020

RN4220  1081 CAAAACTTTGCAGCATGTCGCGCGCTTGTGTCAGAAGGTATTCAACAAGG 1130
TSJY1   1081 CAAAACTTTGCAGCATGTCGCGCGCTTGTGTCAGAAGGTATTCAACAAGG 1130
TSJY2   1081 CAAAACTTTGCAGCATGTCGCGCGCTTGTGTCAGAAGGTATTCAACAAGG 1130
TSJY3   1081 CAAAACTTTGCAGCATATCGCGCGCTTGTGTCAGAAGGTATTCAACAAGG 1130
```

Supplementary Figure 2. Nucleic acid substitutions in the *S. aureus mvaA* gene of temperature-sensitive mutants.

Red letters in the squares indicate the nucleic acid mutations in temperature-sensitive mutants, TSJY1, TSJY2, and TSJY3.

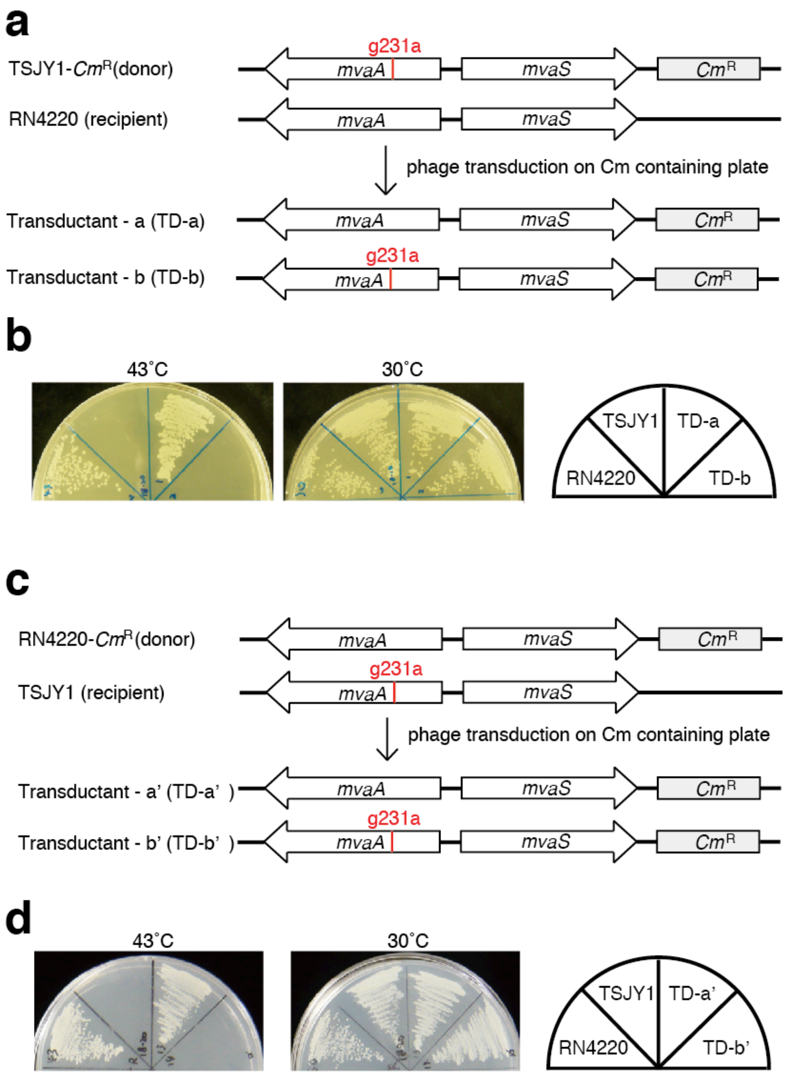

Supplementary Figure 3. Phage transduction experiments to test the relationship between the *mvaA* gene mutations and temperature-sensitive phenotype.

(**a**) Illustration of phage transduction experiments using RN4220 as a recipient and TSJY1 as a donor. The TSJY1 (Cm^r) strain has the chloramphenicol-resistant gene inserted near the *mvaA* gene in the genome. Bacteriophage 80 α was used for the transduction.

(**b**) Overnight cultures of RN4220, TSJY1, and transductants (TD-a and TD-b) were diluted 100-fold, and streaked on LB0 agar plates. The plates were incubated at 30°C or 43°C for 24 h.

(**c**) Illustration of phage transduction experiments using TSJY1 as a recipient and RN4220 as a donor. The RN4220 (Cm^r) strain has the chloramphenicol-resistant gene inserted near the *mvaA* gene in the genome. Bacteriophage 80 α was used for the transduction.

(**d**) Overnight cultures of RN4220, TSJY1, and transductants (TD-a' and TD-b') were diluted to 100-fold, and streaked on LB0 agar plates. The plates were incubated at 30°C or 43°C for 24 h.

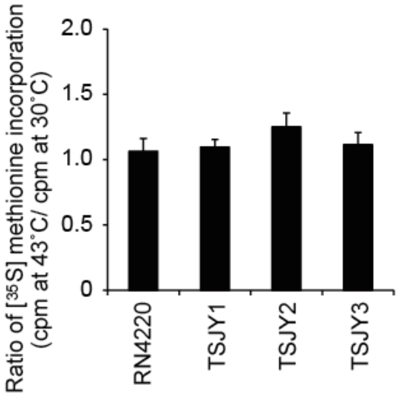

Supplementary Figure 4. Effect of temperature shift on protein synthesis of wild-type and *mvaA* mutants.

Overnight cultures of RN4220 (wild-type), TSJY1, TSJY2, and TSJY3 were diluted 100-fold and further incubated at 30˚C or 43˚C for 5 h. Five-hour incubated cultures were adjusted to OD_{600} = 1.0 in LB0, followed by incubation with [^{35}S]methionine at 30˚C or 43˚C for 15 min, respectively.

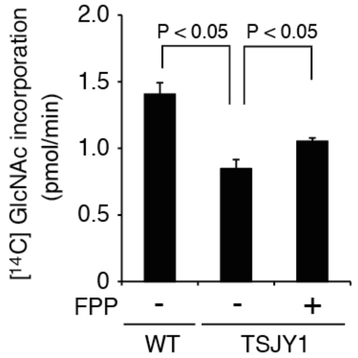

Supplementary Figure 5. Suppression of decrease in peptidoglycan synthesis in the *mvaA* mutant (TSJY1) by the addition of farnesyl diphosphate.

Overnight cultures of RN4220 (wild-type) and TSJY1 were diluted 1000-fold, and further incubated at 43°C. Cultures (OD_{600} = 0.1-0.5) were adjusted to OD_{600} = 0.5 in cell wall synthesis medium (CWSM) containing chloramphenicol (100 μg/ml), followed by incubation with [^{14}C]N-acetyl glucosamine with or without farnesyl diphosphate (100 μM) in CWSM containing chloramphenicol (100 μg/ml) at 43°C for 30 min. Peptidoglycan synthesis was measured by [^{14}C]N-acetyl glucosamine incorporation. FPP: farnesyl diphosphate.

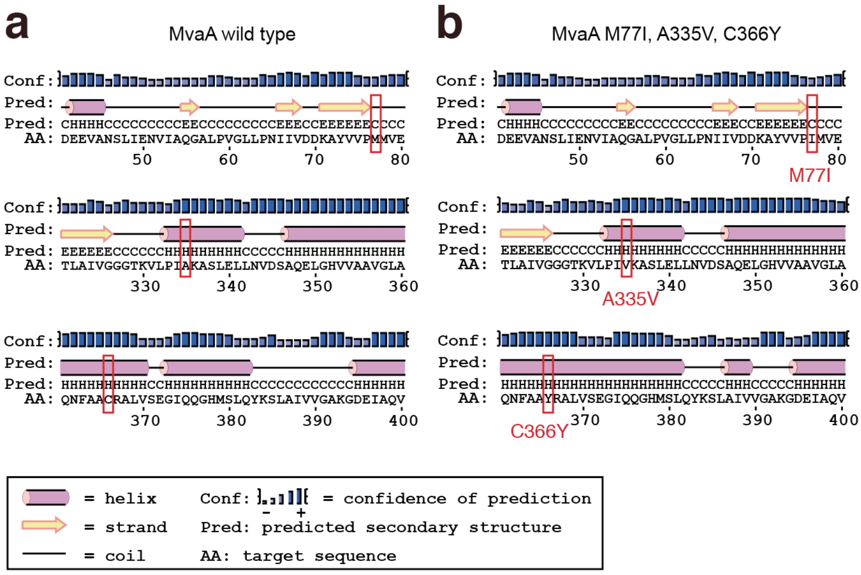

Supplementary Figure 6. Prediction of secondary structures of wild-type, M77I, A335V, and C366Y mutant MvaA protein.

(**a**, **b**) The secondary structures of wild-type (**a**), M77I, A335V, or C366Y mutant MvaA protein (**b**) in *S. aureus* were predicted by PSIPRED v3.0 (http://bioinf.cs.ucl.ac.uk/psipred/) from their primary structures. The amino acid residues of M77, A335, or C366 are indicated by the red square.

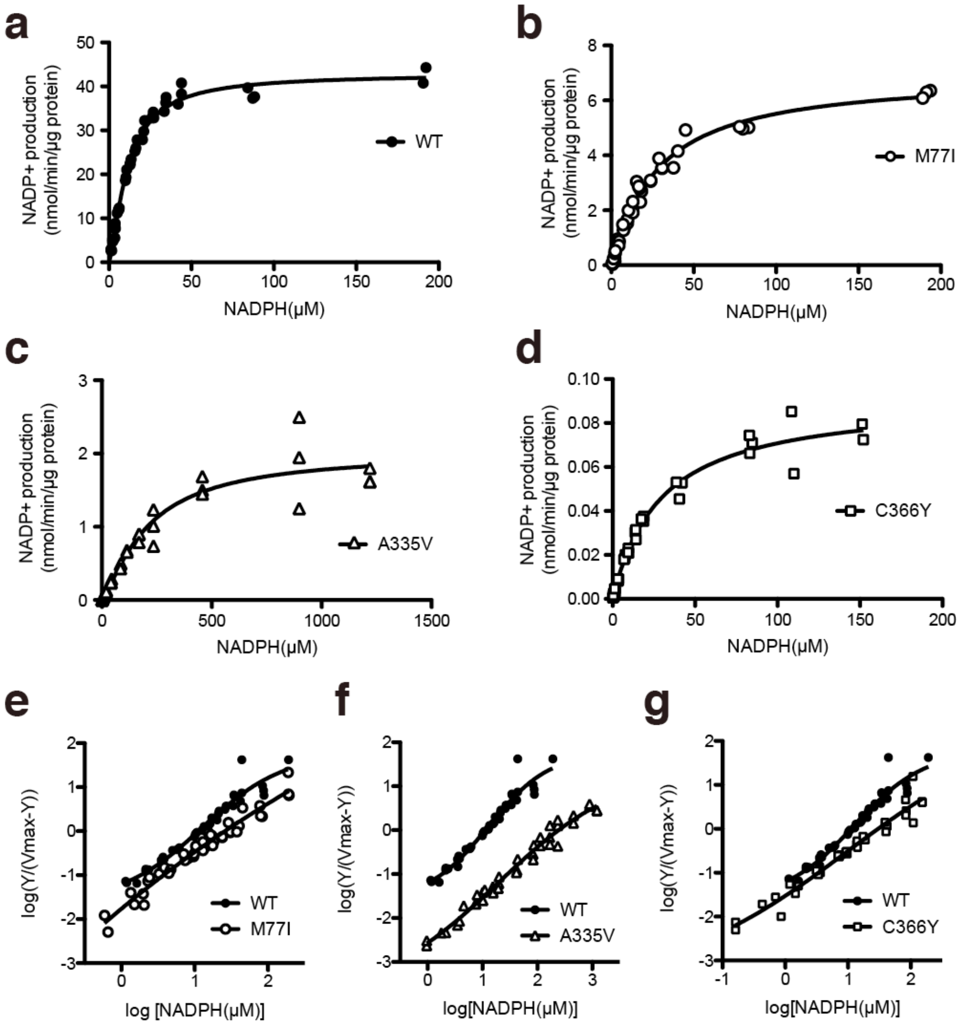

Supplementary Figure 7. Dependency on NADPH concentrations of HMG-CoA reductase reactions by wild-type, M77I, A335V, or C366Y mutant MvaA protein.

(**a-d**) Wild-type, M77I, A335V, or C366Y mutant MvaA proteins were added to a reaction mixture with different concentrations of NADPH. The reaction was performed at 30°C for 30 min. The amount of NADP+ produced in each sample was measured.

(**e-g**) Hill plots for NADPH of HMG-CoA reductase activity of wild-type, M77I, A335V, or C366Y mutant protein.

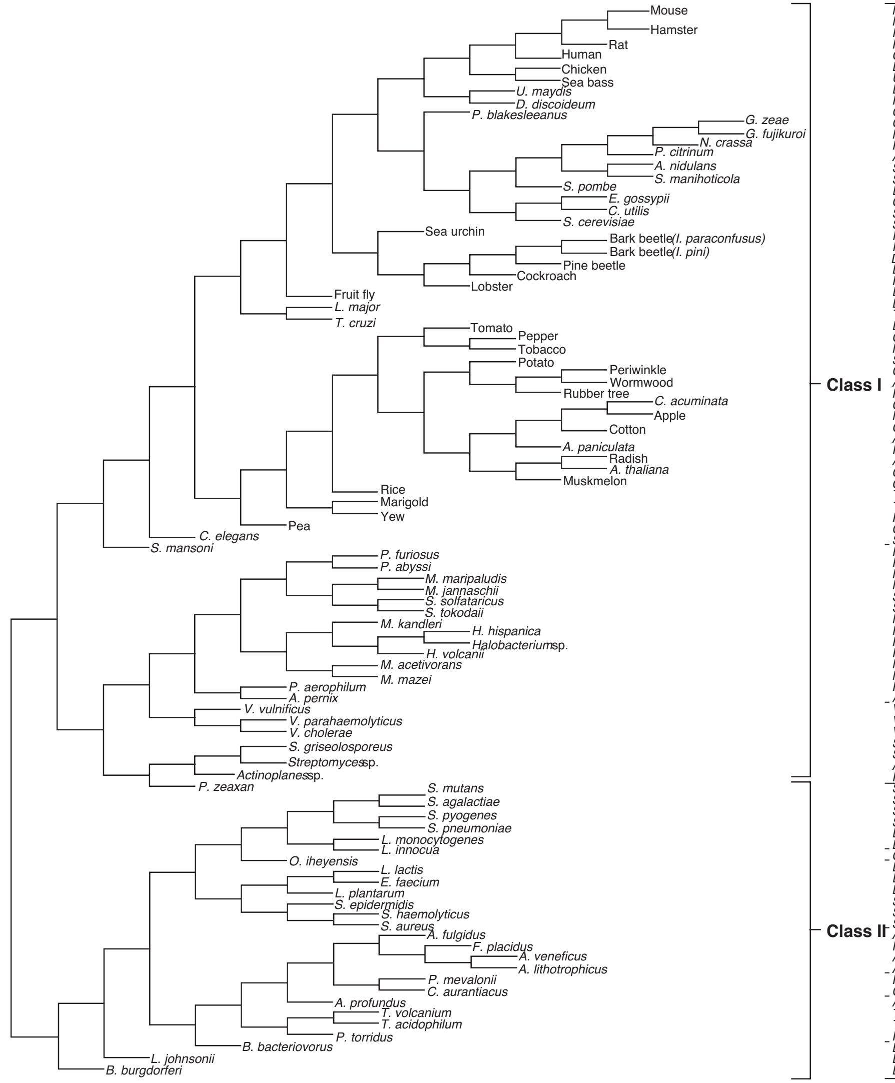

Organism	Kingdom	Corresponding residue to *S. aureus*		
		M77	A335	C366
Mus musculus (Mouse)		M	Q	M
Mesocricetus auratus (Hamster)		M	Q	M
Rattus norvegicus (Rat)		M	Q	M
Homo sapiens (Human)		M	Q	M
Gallus gallus (Chicken)		M	Q	M
Dicentrarchus labrax (Sea bass)		M	Q	M
Ustilago maydis		M	R	M
Dictyostelium discoideum		M	S	M
Phycomyces blakesleeanus		M	Q	C
Gibberella zeae		M	S	C
Gibberella fujikuroi		M	G	C
Neurospora crassa		M	S	C
Penicillium citrinum		M	G	C
Aspergillus nidulans		M	G	C
Sphaceloma manihoticola		M	-	-
Schizosaccharomyces pombe		M	G	C
Eremothecium gossypii		M	S	C
Candida utilis		M	G	V
Saccharomyces cerevisiae		M	G	C
Strongylocentrotus purpuratus (Sea urchin)		M	S	M
Ips paraconfusus (Bark beetle)		M	G	M
Ips pini (Bark beetle)		M	G	M
Dendroctonus jeffreyi (Pine beetle)		M	S	M
Blatella germanica (Cockroach)	Eukaryote	L	A	M
Homarus americanus (Lobster)		M	S	M
Drosophila melanogaster (Fruit fly)		M	S	M
Leishmania major		M	R	L
Trypanosoma cruzi		M	R	M
Lycopersicon esculentum (Tomato)		M	S	M
Capsicum annuum (Pepper)		M	S	M
Nicotinia tabacum (Tobacco)		M	S	M
Solanum tuberosum (Potato)		M	S	M
Catharanthus roseus (Periwinkle)		M	S	M
Artemisia annua (Wormwood)		M	S	M
Hevea brasiliensis (Rubber tree)		M	S	M
Camptotheca acuminata		M	A	M
Malus x domestica (Apple)		M	S	M
Gossypium hirsutum (Cotton)		M	S	M
Andrographis paniculata		M	S	M
Raphanus sativus (Radish)		M	S	M
Arabidopsis thaliana		M	S	M
Cucumis melo (Muskmelon)		M	S	M
Oryza sativa (Rice)		M	A	L
Tagetes erecta (Marigold)		M	S	M
Taxus x media (Yew)		V	A	M
Pisum sativum (Pea)		M	S	M
Caenorhabditis elegans		M	R	M
Schistosoma mansoni		L	R	M
Pyrococcus furiosus		L	R	L
Pyrococcus abyssi		L	R	L
Methanococcus maripaludis		L	R	I
Methanocaldococcus jannaschii		L	K	L
Sulfolobus solfataricus		M	K	L
Sulfolobus tokodaii		L	R	L
Methanopyrus kandleri	Archaea	L	R	V
Haloarcula hispanica		L	F	L
Halobacterium sp.		M	R	L
Haloferax volcanii		L	S	L
Methanosarcina acetivorans		L	R	I
Methanosarcina mazei		L	R	I
Pyrobaculum aerophilum		L	R	L
Aeropyrum pernix		L	R	L
Vibrio vulnificus		L	K	V
Vibrio parahaemolyticus		L	K	V
Vibrio cholerae		L	H	V
Streptomyces griseolosporeus	Eubacteria	L	E	L
Streptomyces sp.		L	E	L
Actinoplanes sp.		L	E	L
Paracoccus zeaxanthinifaciens		L	E	L
Streptococcus mutans		F	V	L
Streptococcus agalactiae		Y	V	L
Streptococcus pyogenes	Eubacteria	F	V	L
Streptococcus pneumoniae		Y	V	L
Listeria monocytogenes		M	A	L
Listeria innocua		M	A	L
Oceanobacillus iheyensis	Archaea	M	A	L
Lactococcus lactis		M	A	L
Enterococcus faecium		M	A	L
Lactobacillus plantarum	Eubacteria	M	A	L
Staphylococcus epidermidis		M	A	C
Staphylococcus haemolyticus		M	A	C
Staphylococcus aureus		M	A	C
Archaeoglobus fulgidus		M	A	L
Ferroglobus placidus	Archaea	M	A	-
Archaeoglobus veneficus		M	A	-
Archaeoglobus lithotrophicus		M	-	-
Pseudomonas mevalonii	Eubacteria	L	A	M
Chloroflexus aurantiacus		-	-	-
Archaeoglobus profundus		M	A	L
Thermoplasma volcanium	Archaea	M	A	V
Thermoplasma acidophilum		M	A	L
Picrophilus torridus		M	S	L
Bdellovibrio bacteriovorus		M	A	L
Lactobacillus johnsonii	Eubacteria	M	V	L
Borrelia burgdorferi		I	S	L

Supplementary Figure 8. Evolutionary tree of HMG-CoA reductase and conservation of M77, A335, and C366 of *S. aureus* MvaA. Phylogenic tree and Kingdom were based on the study of Friesen and Rodwell [4]. Amino acid residues corresponding to M77, A335, and C366 of *S. aureus* MvaA are shown in the right column. Non-conserved residues corresponding to M77, A335, and C366 of *S. aureus* MvaA are indicated in red letters.

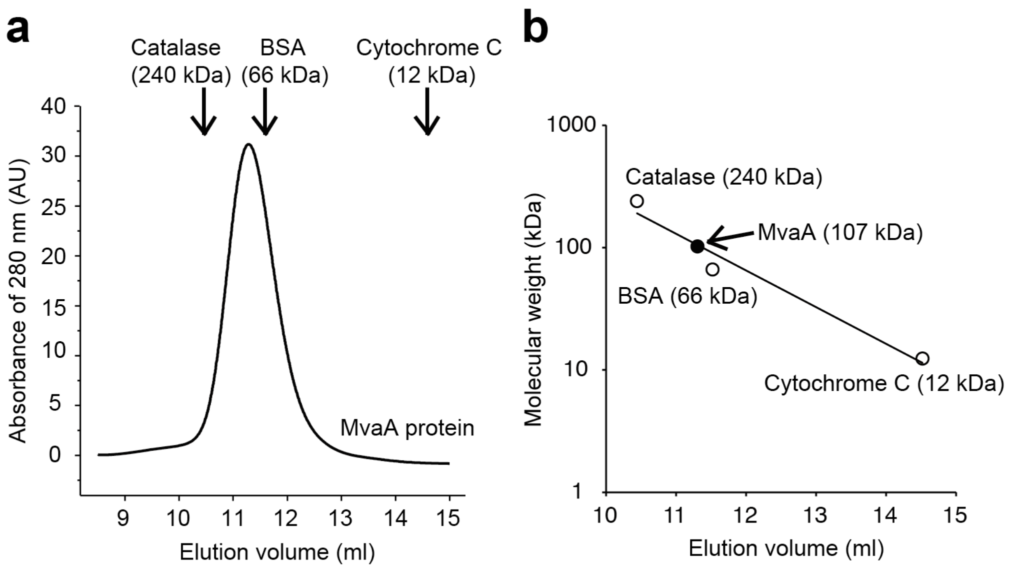

Supplementary Figure 9. Gel filtration of MvaA protein.
(**a**) Purified MvaA protein (330 µg was applied to a Superose 10 column and absorbance at 280 nm was monitored. Peaks of marker proteins (catalase, 240 kDa; bovine serum albumin (BSA), 66 kDa; cytochrome C, 12 kDa) are shown.
(**b**) Estimation of molecular weight of MvaA from the standard curve of the relationship between elution time and peaks of marker proteins (catalase, 240 kDa; BSA, 66 kDa; cytochrome C, 12 kDa).

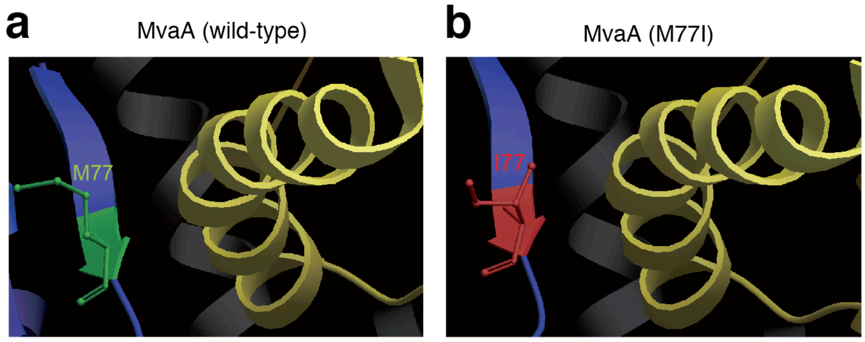

Supplementary Figure 10. Prediction of three-dimensional structures of wild-type and M77I mutant MvaA proteins.

The three-dimensional structures of wild-type (**a**) or M77I mutant MvaA protein (**b**) in *S. aureus* were predicted by RaptorX [5,6] from their primary structures. The structure (PDB ID: 1qaxA) was used as a template. RaptorX is a statistical method for template-based protein modeling [5]. MvaA (wild-type); best template: 1qaxA (p-value < 0.00001), overall uGDT: 351, 425 (100%) residues were modeled, secondary structure: helix (53%), beta-sheet (18%), loop (27%). MvaA (M77I); best template: 1qaxA (p-value < 0.00001), overall uGDT: 350, 425 (100%) residues were modeled, secondary structure: helix (53%), beta-sheet (19%), loop (26%). uGDT is the unnormalized GDT (Global Distance Test) score [5,6]. The amino acid residues of M77(**a**) or I77(**b**) are indicated in green or red, respectively.

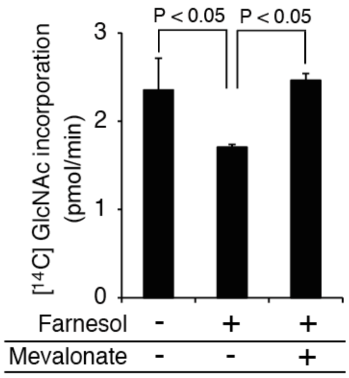

Supplementary Figure 11. Suppression by mevalonate of the inhibitory effect of farnesol on peptidoglycan synthesis of *S. aureus*.

Overnight culture of RN4220 (wild-type) was diluted 1000-fold, and further incubated at 37°C for 5 h. Five-hour incubated cultures were adjusted to OD_{600} = 0.5 in CWSM, followed by incubation with [^{14}C]N-acetyl glucosamine with or without farnesol at 37°C or 43°C for 30 min, respectively. Peptidoglycan synthesis was measured by [^{14}C]N-acetyl glucosamine incorporation.

References

1 Peng, H. L., Novick, R. P., Kreiswirth, B., Kornblum, J. & Schlievert, P. Cloning, characterization, and sequencing of an accessory gene regulator (agr) in Staphylococcus aureus. *Journal of bacteriology* **170**, 4365-4372 (1988).

2 Kaito, C., Morishita, D., Matsumoto, Y., Kurokawa, K. & Sekimizu, K. Novel DNA binding protein SarZ contributes to virulence in Staphylococcus aureus. *Molecular microbiology* **62**, 1601-1617, doi:10.1111/j.1365-2958.2006.05480.x (2006).

3 Ichihashi, N., Kurokawa, K., Matsuo, M., Kaito, C. & Sekimizu, K. Inhibitory effects of basic or neutral phospholipid on acidic phospholipid-mediated dissociation of adenine nucleotide bound to DnaA protein, the initiator of chromosomal DNA replication. *The Journal of biological chemistry* **278**, 28778-28786, doi:10.1074/jbc.M212202200 (2003).

4 Friesen, J. A. & Rodwell, V. W. The 3-hydroxy-3-methylglutaryl coenzyme-A (HMG-CoA) reductases. *Genome biology* **5**, 248, doi:10.1186/gb-2004-5-11-248 (2004).

5 Peng, J. & Xu, J. RaptorX: exploiting structure information for protein alignment by statistical inference. *Proteins* **79 Suppl 10**, 161-171, doi:10.1002/prot.23175 (2011).

6 Kallberg, M. *et al.* Template-based protein structure modeling using the RaptorX web server. *Nat Protoc* **7**, 1511-1522, doi:10.1038/nprot.2012.085 (2012).
